# On the "Mal d'Estomac."

**Published:** 1822-06

**Authors:** Wm. Telford

**Affiliations:** Surgeon, R.N.


### PATHOLOGY.

### ART. III.—*On the "Mal d'Estomac."* By WM. TELFORD, Surgeon, R.N.

THIS disease most generally appears among slaves recently purchased, and soon after their masters have left off feeding them daily, but let them trust to their gardens for the chief part of their food; only allowing them seven or eight salted herrings a-week, (which are sometimes half rotten,) or perhaps salted fish or beef. The weakly, whether from constitution or being too soon turned out to duty after sickness; the indolent and dirty, that neither cultivate their grounds so as to be supplied with plenty of fresh vegetables, or to have their houses in good order; those that are employed, during crop, in the rum-cellars, where the air is, in general, damp; and all other slaves living on estates much exposed to the variations of the weather, are very subject to this complaint. The negroes are, *cæteris paribus*, more apt to be attacked with it than others.

It hath very frequently been observed, in those who have recovered from the disease, that, from being again exposed to the remote causes, they are again suddenly attacked with it in a most violent manner; and here, I think, I have remarked the progress of the disease to be much quicker than on its first attack; when, also, the difficulty of breathing is the most troublesome complaint. This frequently happens when the rains set in; and I have also seen it arise from a negro's being put into confinement for some time. This obviously points out the necessity of watching the first appearance of the disease; and also points out the use of prophylactics when the remote causes prevail.

The disease hath been known to recur very frequently in the same person; though persons, who have been once cured of the disease, have remained free from it a[illegible] their lives. As the disease is very different in its progress, so is its duration, whether it terminates in life or death.

I have had a patient, in whom the disease proved fatal on the fourteenth day, in spite of every remedy that could be given. The weather was wet, and the situation very cold for the island. He had been long known to eat clay; but, on his being suddenly attacked with a difficulty in breathing, and a swelling in the extremities, which increased very fast, he was suspected to have eaten something poisonous. Emetics, cathartics, diuretics, with frictions, &c. were immediately had recourse to; and, notwithstanding all these evacuants, the swelling increased. Blisters and punctures, to relieve the immediate tension of the skin, were also resorted to; but with no better success. Bark, wine, and steel, were given; but the swelling still increased, till at length his whole skin was put quite on the stretch, and he died, absolutely calling for victuals, and in the full enjoyment of all his intellects. I saw him a few hours before his death; and another of my patients, at considerable distance, requiring all my attention, I was not able to return in time to examine the body.

DISSECTION I.

A negro man, in the prime of life, stout, well made, and a very valuable tradesman, had, on the day and night preceding, been upon a neighbouring estate to see his wife. Returning home very early on Monday morning, told the manager that he felt a stricture about the stomach, which prevented his breathing; and, during the time the manager was questioning him, expired without a struggle. I was immediately sent for, and arrived in about twenty minutes. I could find no mark of external injury; but was told he had a slight attack of the mal d'estomac for some short time, and was under a course of *tisanne*, made by the hospital nurse.

I obtained leave to examine the body, which was still warm. On opening the abdomen, the quantity of fluid did not strike me as considerable; but, in the thorax, I found the pericardium very much distended with a watery fluid, which being absorbed with a sponge, enabled me to look into the right cavity, where I observed a very considerable quantity of clear fluid, and swimming in it, as well as adhering in some parts to the pleura, several films of coagulable lymph. Some of them were at least two inches in length, filled with air, and in the shape of a pear.

On the left side, appearances were exactly similar. The lungs seemed to have undergone a very considerable degree of compression, and, from their figure in their upper part, must have forcibly been pushed up under the first rib: their internal structure appeared sound. The heart was of a very large size, and its texture soft. The right auricle and ventricle were filled with blood, partly grumous and partly fluid. The liver, and other abdominal viscera, appeared natural. The stomach contained about a quart of thin watery fluid, with the remainder of a few leaves of vegetables in a half-dissolved state; and the inner coat of the stomach, about the pylorus, was jellied.

The brain appeared flaccid, and very few of the blood-vessels contained red blood; the lateral ventricles, also the third and fourth, contained but little fluid. The corpora striata, plexus choroides, thalami nervorum opticorum, tubercula quadragemina, and cerebellum, appeared in their natural state; and I could not discover any thing here that could be suspected to have produced death. May we, then, suppose the water in the thorax to have occasioned his sudden death? I suspected it; and, this being the first I had ever examined (that died of this disease), I determined, in future, to observe very carefully the progress and degree of the affection in the breast.

DISSECTION II.

About five months after the foregoing, I was called upon to examine the body of a negro who had died of the mal d'estomac, upon an estate where I had not the common attendance. The history they gave me of his disease was nearly the following: —He was a new negro, and, ever since purchased, appeared dull, heavy, and torpid; his features naturally large, forehead flat, eyes small and hollow. Whenever he walked up hill, or was put to hard work, he complained of weakness, especially about the præcordia; respiration quick, very laborious; and great palpitation of the heart.

No swelling had appeared in his lower extremities till a few days before his death; and, during the whole course of his disease, his appetite was good and digestion quick.

From the first appearance of the disease he had drank a ptisan, which, in numberless instances, had succeeded in others, but in him no sensible operation or alteration was observed. By the advice of a gentleman of the faculty, he drank a strong hydragogue medicine, which purged him very briskly; but afterwards the swelling increased very fast.* That, after eating a very hearty supper, and appearing nearly in his usual state, he suddenly complained of faintness: some wine was given to drink, but he expired in a few minutes without any struggle.

Before opening the body, I observed the face, trunk, abdomen, scrotum, and inferior extremities, considerably swelled.

In making an incision from the top of the sternum to the navel, the tela cellulosa contained a considerable quantity of water; and the tela adiposa was filled with fat of a deep citron colour.

In the cavity of the abdomen floated a considerable quantity of watery fluid, of a yellowish colour; the omentum was of a deep citron colour; and the liver of an enormous size, and its colour light. Nearly the whole of its convex surface adhered to the diaphragm and peritoneum lining the false ribs. Its lower edge, the cellular substance, and surface of the right kidney, were in a state of inflammation, (the French word *engorgement*, perhaps, will convey a better idea of it than any word in our language,) of a deep red, tending to a livid colour.

On cutting into the substance of the liver, no abscess could be discovered; but there issued out an astonishing quantity of bloody water, so fluid that it ran in a full stream through a male catheter, (that I introduced into one of the branches of the vena cava,) and to a considerable distance from its end. I suspected there might be a cyst formed somewhere; but, on cutting along the catheter, and through all the concave part of the liver, no such cyst could be discovered. The quantity of water was so considerable as to astonish me, and I waited with patience until it had stopped; when the size of the organ was diminished to even a smaller size than might have been expected in a lad of his stature.

The gall-bladder was filled with bile, of a dilute yellow co-

* Ever since the commencement of the swelling, he had always complained of a pain, and pointed to the right hypochondria as its seat.

4

lour; the spleen, stomach, and other viscera, sound; the bladder empty and rugated.

In the cavity of the thorax, on each side, was found about half a pint of thin fluid, tinged with yellow. The lungs were white and as if long soaked in water, and pushed up under the first rib. The heart was very large, and the pericardium much distended with a fluid of the same colour, &c. as that in the thorax. In the right auricle and ventricle were found small masses of coagulated lymph, adhering to the sides by small filaments. The substance of the heart was so much relaxed, as easily to admit the finger to be pushed through the ventricle.

In this case I did not inspect the brain.

DISSECTION III.

A new negro, remarkably stout and well made, received a blow upon the top of the left shoulder, which was, seemingly, so insignificant as to pass unnoticed for a considerable time, although a swelling ensued, with considerable pain, inflammation, &c. &c.; which symptoms were all placed to the account of Guinea worms, several of them having been extracted from various parts of his body. The swelling subsided on the top of the shoulder, but was succeeded by a very considerable one in the arm-pit, which I was called in to examine. They furnished me with the foregoing history, concealing only the circumstance of the blow preceding the first swelling.

The tumor in the arm-pit broke of itself, and discharged plentifully; but no appearance of worm taking place, getting at the knowledge of the blow, and the discharge becoming very considerable and ill-conditioned, I enlarged the opening. Appearances were not at all bettered by this; the discharge became extremely fetid; the patient's arm, and all his left side down to the toes, became anasarcous, and his general health very much impaired, notwithstanding he took large quantities of bark and such other medicines, and nourishing diet with wine. I had now no doubt that an abscess had formed within the capsular ligament of the joint, and suspected that the ends of the bones might be diseased. By enlarging the opening in the axilla, I discovered the opening in the capsular ligament, which induced me immediately to set about the operation of amputating the arm at the shoulder-joint. This I performed on the 5th of March; and, by saving a very large flap, from the deltoid muscle, the extremity of the scapula (which bone appeared perfectly sound,) was covered, and the size of the wound reduced very considerably.*

* The head of the os humeris was quite carious, of a black colour, and extremely fetid. The tela cellulosa of the arm, fore-arm, hand, and fingers, was

From this time, he got strength, vigour, and appetite, daily; the wound looked extremely well, discharged a moderate quantity of pus, and the healing process went forward fully as well as my most sanguine hopes could wish. He took large quantities of bark and port-wine, and I ordered him to have food five or six times a-day. On the 20th of April, the wound was not three inches in length, and very little more than one in breadth. He appeared dull for some days before; his face, eyelids, and neck, became a little swelled, without my being able to assign any reason. Steel, with confectio cardiaca, ginger, oil of mint, and red bark, was put in the room of the pale, and were now used, with an additional quantity of port-wine, and strict orders respecting his nourishment. He continued languid, and the swelling, with difficulty of respiration, increased very fast; and one morning, on going into the hospital to dress him, I found him in a swoon, from which he with some difficulty recovered. This led to a particular inquiry respecting his food and medicines; when, by the evidence of one of his countrymen, we found out that the hospital-keeper, instead of giving him the victuals allowed for him, applied them to another purpose; and that, in fact, his dejection, weakness, and fainting, had been the sole effect of inanition. This took place on the 25th of April; and, by a very particular attention to his food and medicine, he had, in eight days, very considerably recovered his strength. During all this period the wound made no progress in healing; but, on the contrary, a considerable eruption of small miliary pustules took place round its edges, which produced little ulcers. The discharge, instead of thick benign pus, became quite serous and nearly transparent, hardly discolouring the dressings.

On the 4th of May, he was seized with a paroxysm of intermittent fever, which returned regularly every forenoon; and the cold fit would sometimes continue upon him until five and six o'clock in the afternoon. He again became weaker, refused food; and, on the 10th, he died in the cold fit, about four in the afternoon.

On opening the abdomen, a very considerable quantity of fluid, of a greenish colour, and considerably fetid, was found within the peritoneum; which, on exposure to the air and sun, did not coagulate. I was prevented from making any experiment with acids or ardent spirits, from the inattention of my servant, who threw it away next day in my absence. The heart was of the natural size, nor contained in its cavities any

---

distended with a transparent jelly; which, I have no doubt, was the lymph coagulated in its vessels and cells: exactly what Dr. Hendy mentions to take place in the glandular disease of Barbadoes.

appearance of polypi. In the ascending cava, a very slight coagulation of the blood had taken place. The lungs were of a purple colour; and each cavity of the thorax contained upwards of half a pint of the green-coloured fluid observed in the abdomen; as had also the pericardium, which was very much distended by it.

The preceding history of dissections plainly points out the seat of the disease, and also some of its causes. In every one we have found effusion in the chest, pericardium, and (except in the first case) in the abdomen, with less or more of anasarca. In the cases formerly mentioned there was a very considerable degree of anasarca. In one patient, that I treated with success, the anasarca was accompanied by ascites; and, by the operation of tapping, I drew away, from the cavity of the abdomen, at least three gallons of water.

In another case which fell under my observation, the affection of the chest was the first complaint, and was very soon followed by a general anasarca. In this case, there was a short troublesome cough; the oppression in the breast was such as to threaten immediate suffocation; the anxiety at the præcordia was almost insufferable; and the palpitation of the heart so violent, that it could be observed at a very considerable distance. The only means of relief the man found was in vomiting; and here I thought a very evident preference was due to blue vitriol over the antimonial preparations. After repeated and very copious evacuations, both by vomit and stool, the symptoms were a little relieved; and the cure was afterwards perfected by red bark, rust of steel prepared, and aromatics.

In no disease whatever have I observed the superiority of red bark over pale more notoriously than in this: but these remarks are out of place, and will come in better under the method of cure.

With respect to a definition of this disease, it may be gathered from an enumeration of the symptoms; and the appearances of the symptoms may be satisfactorily accounted for from the appearances on dissection: but where to find a just distinction between this disease, anasarca, and hydrothorax, I am really at a loss; and will, therefore, leave it to the penetration of my readers to draw such distinctions as they think proper.

In regard to the method of cure, I have very little new matter to bring forward. Except in the one instance mentioned in the beginning, I have never failed. Though frequently puzzled, I have still been able to bring it about; although I have always dreaded a relapse.

The disease occurred, very lately, in a negro-boy of my own, a creole, aged about fourteen or fifteen years. From some cause of chagrin that I have not been able to find out, he became quite dull, heavy, and sleepy; he seemed to shun the society of the other domestics; and, whenever he was in a situation where he could lay down, fell immediately asleep, and frequently, in this situation, would sleep the whole night out of doors.

I observed that, in following me on horseback, though at an easy pace, he had an extreme difficulty of breathing, with great palpitation of the heart: this, when at his ease, was accompanied with a short cough. Soon afterwards, I observed his face a little swelled, his belly rather enlarged; and to these succeeded a swelling of the feet, with enlargement and pain in the lymphatic glands in the groin.* His sleep now became disturbed; and he annoyed every person within hearing, from the noise that he made, which resembled a person suffocating. I gave him no medicine, until the difficulty of breathing was such that every muscle, any way connected with that function, was in action to perform it.

I gave him, by spoonfuls, a mixture composed of jalap, cream of tartar, and tartar emetic: this he took every two hours through the day, which operated very powerfully, both by vomit and stool. The two succeeding days, he took, every four hours, a tea-spoonful of an electuary, composed of red bark, rust of steel, powdered ginger, and cordial confection: this purged him every day at least five or six times, and he was evidently better.

On the fourth day, he took half a grain of blue vitriol every two hours, until he had taken five doses: this puked him also, though not a great deal; but he complained that it produced a most uneasy sensation in the stomach, and purged away an astonishing quantity of water.

On the fifth, appearances were very much mended; and he again, for two days, took his electuary. The swelling of his feet was now quite gone, the skin wrinkled; and he breathed tolerably freely.

On the seventh, I repeated the blue-vitriol mixture three times, which produced an effect similar to the first; and since that I have had no occasion to give him any medicine. He is daily mending, and at present nearly in as good health as ever.

The foregoing is the general method of treatment, which must be varied according to circumstances of constitution, period of the disease, &c. I have always found evident good effects from brisk vomiting and purging on the onset, and im-

* From his indolent disposition, a vast number of the insects called *chiegoes* had got into his feet, which frequently produce a swelling and inflammation of these glands.

mediately after to ply them hard with the electuary of red bark and steel, &c. &c. Great assistance is also derived from diet: the most nutritive, with a free use of seasoning and generous wine, is to be preferred. At the same time, clothing, gentle exercise, frictions, &c. are not to be omitted. There is a popular mistake respecting exercise, which sometimes, I am informed, hastens the patient's death,—i. e. forcing them to perform what is really beyond their strength. In these exertions, negroes have sometimes dropt down dead.

There is another error, which planters are very apt to fall into,—viz. turning negroes out to work before their strength is absolutely restored: this often occasions a relapse, and in one instance proved fatal under my own eye.

Every planter almost judges himself possessed of medical skill sufficient to conduct the common diseases on estates; and, perhaps, in no instance does it oftener occur than in that of turning negroes out of the hospital before they are recovered, that we have the mortification to find our opinions thwarted, and our advice contemned. It is the common cry, "Give medicines to strengthen them," when rest and good nourishment would suit them better.

I think I have seen advantage from some of the bitters (i. e. the herbs and roots,) of the country, infused in rum, and a wine-glass full given every morning. Others find great advantage from blue vitriol given in rum every morning; and, again, some are fond of ptisans; the best of which appear to me to be composed of bitters, aromatics, astringents, stimulants (of the acrid kind), and a sufficient proportion of some of the farinacea, or, what answers equally well, a quantity of molasses, so as to produce a brisk fermentation; and then some iron is generally added. These, when properly made, are far from unpleasant, and by no means to be esteemed despicable medicines.

A change of air, from a moist and cold to a dry and warm situation, is useful; and, very often, going to sea has saved the patient's life: but it is necessary to observe that this is often too long delayed. This would appear particularly useful when they have such a propensity to eat clay or dirt, that no fear of punishment can make them resist the temptation: but on-board, unless looked after, they will eat ashes.

Having thus enumerated the chief particulars of the disease, so far as I know, I must leave my readers to draw their conclusions.

